# Measurement and simulation of irrigation performance in continuous and surge furrow irrigation using WinSRFR and SIRMOD models

**DOI:** 10.1038/s41598-023-32842-8

**Published:** 2023-04-08

**Authors:** Mojgan Radmanesh, Seyed Hamid Ahmadi, Ali Reza Sepaskhah

**Affiliations:** 1 Water Engineering Department, School of Agriculture, Shiraz University, Shiraz, Iran; 2 Drought Research Center, Shiraz University, Shiraz, Iran

**Keywords:** Environmental sciences, Hydrology

## Abstract

The SIRMOD and WinSRFR models were used to model and assess the irrigation performance under continuous and surge irrigation strategies with two furrow lengths of 70 m and 90 m and stream sizes of 0.4 l/s and 0.6 l/s for each length. According to the normalized root mean squared error (NRMSE) and the relative error (RE), WinSRFR had, on average, excellent accuracy in the continuous and surge irrigation for simulating advance-recession times (NRMSE: 6.15 and 4.24% for advance time, and 2.20 and 5.20% for recession time), infiltrated water depth (NRMSE: 3.37 and 6.38%), and runoff volume (RE: 6.93 and 2.57%), respectively. SIRMOD had also, on average, excellent simulation in the continuous and surge irrigation for advance-recession times (NRMSE: 3.34 and 2.45% for advance time, and 2.28 and 6.41% for recession time), infiltrated water depth (NRMSE: 2.98 and 5.27%), and runoff volume (RE: 5.31 and 17.49%), respectively. The average of irrigation application efficiency (*AE*), distribution uniformity (*DU*), deep percolation (*DP*), and tail-water ratio (*TWR*) were 61.50, 90.25, 11.75, and 26.75% in continuous irrigation, and 72.03, 94.09, 8.39, and 19.57% in surge irrigation, respectively, which shows that surge irrigation increased *AE* (irrigation management performance) and *DU* (irrigation method performance) and reduced *DP* and *TWR* compared to continuous irrigation*.* Moreover, longer furrow lengths increased *AE* and *DP* under both irrigation methods, while it decreased *TWR* and *DU*. However, increasing the stream size decreased *AE* and *DP* and increased *TWR* under both continuous and surge irrigations. The higher stream size improved *DU* in continuous irrigation but reduced *DU* in surge irrigation. The results confirmed that both SIRMOD and WinSRFR are reliable analytical tools to evaluate furrow irrigation strategies for improving irrigation management. In conclusion, this study showed that surface irrigation models could be employed in practice by irrigation engineers and practitioners to design and define the optimized furrow length and stream size in arid and semi-arid areas where efficient and high performance irrigation strategies are required to save water and reduce water loss.

## Introduction

Surface irrigation is still the most popular irrigation system because of its simplicity of design, low cost and energy requirements, and low investment requirements^1,2^. Currently, more than 70% of the irrigated lands in Iran are under surface irrigation, and most of these fields are irrigated as furrow irrigation^3^. However, the sustainability of surface irrigation systems does not solely depend on irrigation technology but also depends on proper irrigation management^4^. The challenges for adopting climate resilient irrigation practices are still enormous because more than 95 and 80% of irrigated farms in Asia and the Middle East are surface irrigated^4^. Therefore, this makes surface irrigation an important target research goal since it has great impact on the catchment water resources in semi-arid areas^5^. In this regard, precise surface irrigation management depends on designing appropriate surface irrigation methods, which improve irrigation performance indicators^6,7^. In a recent study, Bryant et al.^8^ reported that nearly 80% of mid-southern U.S. farms are irrigated through furrow irrigation with low application efficiency. Thus, improving the performance of surface irrigation systems requires extensive research and evaluating the deriving components of the irrigation system^4,9^.

Generally, imprecise irrigation management would increases runoff and deep percolation in furrow irrigation system and reduces the efficiency and distribution uniformity^10^. The primary parameters that influence irrigation system performance are stream size, furrow field length, soil roughness, field slope, soil infiltration rate, and cut-off time^4^. To reduce water losses and increasing the efficiency of surface irrigation, researchers have suggested surge irrigation and cutback flow methods^11,12^, appropriate field length and slope^13,14^, and determining the appropriate amount of stream size and cut-off time^13,15^.

Surge irrigation is the process of intermittently applying water to furrows in a series of nearly short on- and off-times that could subsequently change physical characteristics of the soil surface resulting in uniform application and infiltration of water^16–19^. The first studies of surge irrigation dates back to early 1980 with focus on improving advance rate, surface irrigation efficiencies and uniformities and reducing the total amount of irrigation water^20–23^. Given that continuous irrigation has low efficiency^24^, the use of surge irrigation combined with appropriate furrow variables (stream size, cut-off time, and furrow length) would be a promising alternative to continuous irrigation for conservation of water resources and reduction of water losses^12,25,26^. Surge irrigation has been reported to reduce water losses and improved water distribution in soil^11,18,19,27,28^. Nevertheless, Henry et al.^29^ reported that about 4–6% of furrow-irrigated fields are just irrigated by surge irrigation, which is very small compared with the continuous irrigation. In a recent study, Bryant et al.^8^ reported that surge irrigation can lead to an increase in application efficiency up to 209% while runoff, deep percolation, and total water applied were reduced by 57, 64, and 31%, respectively. Coolidge et al.^30^ showed that surge irrigation reduces the infiltrated volume for the advance time and improves the distribution uniformity along the furrow.

However, the hydraulic process of surface irrigation is complex since it simultaneously combines surface flow with infiltration into the soil profile. Several computer models have been developed for surface irrigation simulation. The use of models in design allows taking into consideration the factors that interact with multicriteria analysis of surface irrigation^4^. The WinSRFR and SIRMOD models are among the most powerful models used to design and evaluate surface irrigation systems in continuous and surge irrigation methods. Xu et al.^14^, Nie et al.^31^, and Mazarei et al.^2^ used the WinSRFR model to evaluate and optimize the physical parameters of furrows. Likewise, various researchers^32–34^ used the SIRMOD model to evaluate continuous surface irrigation systems whilst Ismail et al.^35^, and Ismail and Depeweg^11^ used the SIRMOD model in assessing surge irrigation.. However, because of the difficulty in implementing and managing surge irrigation, few studies have been done on surge surface irrigation.

Previous studies have focused on evaluation and optimization of the continuous surface irrigation systems^2,13,14,36^. However, there is a knowledge gap on evaluation and simulation of surge irrigation management. In this study, different surge irrigation scenarios have been developed and simulated by the SIRMOD and WinSRFR models. To the best of our knowledge, there is no previous study comparing the performance of these two models in surge irrigation, and this study provides solutions to simplify surge irrigation simulation. SIRMOD and WinSRFR models potentially serve as the analytical tools for simulating field irrigation management scenarios that help decision-makers, field managers, and irrigation professionals for assessing furrow irrigation techniques. The concern considered in this research is the furrow irrigation performance and the reduction of water losses; thus, efforts have been made to improve them. We hypothesized that surge irrigation improves irrigation performance. The study aimed to assess the performance of the two simulation models namely; SIRMOD and WinSRFR under continuous and surge irrigation. Thus, the specific objectives of this study were: (1) evaluating continuous and surge irrigation methods under field conditions to improve irrigation performance, and (2) assessing model performance of the SIRMOD and WinSRFR models in simulating the continuous and surge irrigation methods.

## Material and methods

### Field description

**Figure 1:**
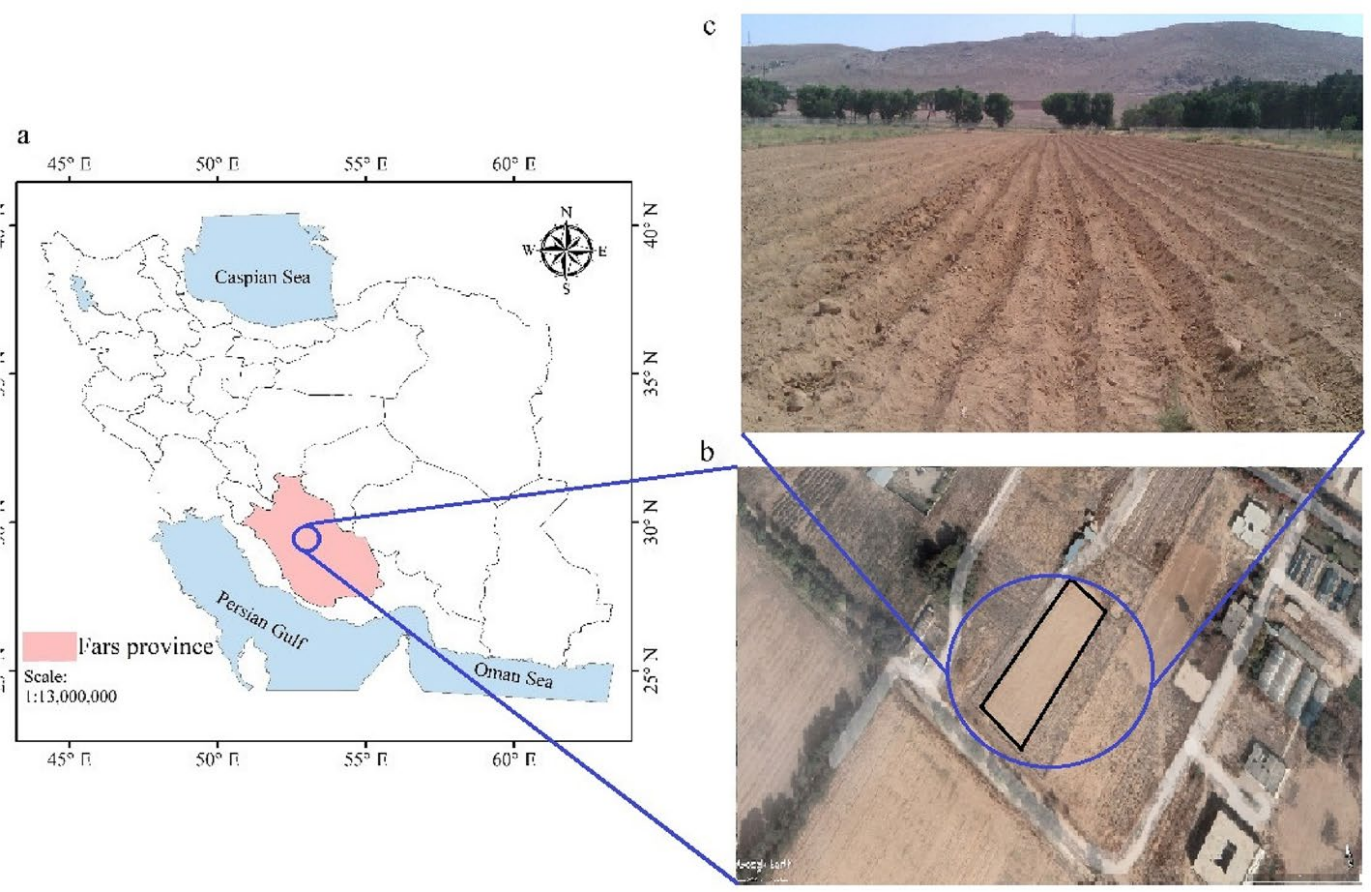
Location of the case study area at the School of Agriculture, Shiraz University, Shiraz, Iran. (**a**) is producd by the open sourse QGIS version 3.24 (www.qgis.org); (**b**) is deveopled using the Google Earth Pro version 7.3.6.9345 (https://earth.google.com/web/).

The field experiment was conducted at the School of Agriculture, Shiraz University, Shiraz, Iran. The location of the field experiment was 52°32′N, 29°36′E and 1810 above m.s.l. in summer 2016 (Fig. 1). The experimental field has a deep and uniform silty clay loam soil texture. Experiments were carried out in furrows with lengths of 70 m (L_1_) and 90 m (L_2_), furrow width of 0.75 m, and longitudinal slope of 0.002. It should be noted all field experiments were conducted on the bare soil under open-end boundary conditions, and there is a buffer furrow between experimental furrows. The uniform and constant stream sizes of 0.4 and 0.6 l/s were supplied by a typical residential water meter (ISO4064, Brass, ½ inch inlet size, and measuring accuracy of ± 0.1 L at 5–40 °C).

### Irrigation methods and practices

Two stream sizes of 0.4 l/s (Q_1_) and 0.6 l/s (Q_2_) were used to irrigate the furrows at constant rates. The furrow lengths and stream sizes were chosen based on the standard local layout of small farms in the region^37^. Furthermore, preliminary trials showed that higher stream sizes induced soil erosion in the furrows. The Washington State College (WSC) flume type 1 was installed at the end of the furrows to measure runoff volume (tail-water). The required irrigation depth was considered 0.05 m to ensure uniform seed germination and crop establishment according to the current practices in the region recommended by the Office of Farm Management, School of Agriculture.

Field data was collected under continuous and surge irrigation. The continuous irrigation was the reference irrigation method with which surge irrigation was compared for irrigation performance subject to the combinations of furrow length, stream size, and cycle ratio as the irrigation treatments. The on-time and off-time of the surge flows were 8 min and 8 min, respectively, which resulted in cycle time of 16 min (CT) and cycle ratio (CR) of 0.5 based on Eqs. (1) and (2). The on- and off-times were selected based on a set of preliminary field tests so that at least three surges could be applied to complete the advance phase of the irrigation in the furrows.

1 $$CT = on - time + off - time$$

2 $$CR = \frac{on - time}{{CR}}$$

To determine the advance-recession times along the furrows, the length of all furrows were subdivided into 5 m intervals (stations) and the advance-recession times were monitored and recorded at all stations. Infiltrated water depth over the soil profile was measured by monitoring soil water content (SWC). The SWC was gravimetrically measured using a metal spiral auger down to 100 cm. The initial SWC was measured before starting the experiments. Having done the experiments and 48 h after each irrigation event, the soil samples were collected at 20 cm intervals over soil profile at five soil depths as 0–20, 20–40, 40–60, 60–80, and 80–100 cm at five selected stations (0, 15, 35, 60 and 70 m) and at seven selected stations (0, 15, 30, 50, 60, 80 and 90 m) along the 70 m and 90 m furrow lengths, respectively. The soil sampled were immediately weighted at the field (wet weight) and then were oven-dried at 105 °C for 24 h (dry weight) to calculate the gravimetric SWC^38^. The volumetric SWC was calculated by multiplying the soil bulk density (Table 1) by the gravimetric SWC. The volumetric SWC was then used to calculate the depth of infiltered water through multiplication with the associated soil depth (20 cm). Table 2 summarizes the field measurement data.

**Table 1:** Physical properties of the soil at the experimental site^87^.

Properties	Soil depth (cm)		
	0–30	30–60	60–90
FC (%)	32	33	35
PWP(%)	17	19	19
$\rho_{b} \;({\text{g}}\;{\text{cm}}^{ - 3} )$	1.4	1.5	1.5
Sand (%)	11	10	16
Silt (%)	56	51	50
Clay (%)	33	39	34

**Table 2:** Field characteristics information of the combination of furrow length and stream sizes under the continuous and surge irrigations.

Parameters**	Continuous irrigation				Surge irrigation*			
	Q_1_L_1_	Q_1_L_2_	Q_2_L_1_	Q_2_L_2_	Q_1_L_1_R_0.5_	Q_1_L_2_R_0.5_	Q_2_L_1_R_0.5_	Q_2_L_2_R_0.5_
Q (l/s)	0.40	0.40	0.60	0.60	0.40	0.40	0.60	0.60
L (m)	70.00	90.00	70.00	90.00	70.00	90.00	70.00	90.00
CR (-)	–	–	–	–	0.50	0.50	0.50	0.50
T_co_ (min)	174	196	156	147	198	247	153	198
V_in_ (m^3^)	4.17	4.70	5.62	5.30	4.23	4.57	4.93	5.96

*Cycle time is 16 min in surge irrigation (8 min on-time + 8 min off-time).

**Q: stream size; L: furrow length; CR: cycle ratio in surge irrigation; V_in_: volume of applied water; Tco: Cut-off time; R_0.5_: surge irrigation with cycle ratio of 0.5.

### Infiltration equations

Infiltration is one of the most sensitive hydraulic parameters affecting surface irrigation, and is of course one of the most difficult parameters to be determined in the field ^31^. The Kostiakov-Lewis equation is the widely used equations in determining infiltration parameters as:

3 $$Z = kt^{\alpha } + f_{0} t$$

where *Z* is the cumulative infiltration (m^3^m^−1^), *t* is the opportunity time of infiltration (min), *f*_0_ is the basic infiltration rate (m^3^m^−1^ min^−1^), and *k* and *a* are empirical coefficients (*a:* dimensionless, *k:* m^3^ min^−α^ m^−1^).

The infiltration parameters of the Kostiakov-Lewis were determined using the two-point method of Elliott and Walker^39^. The base infiltration rate ${f}_{0}$ is determined according to Walker and Skogerboe^40^ as follows:

4 $$f_{0} = \frac{{Q_{in} - Q_{out} }}{L}$$

where Q_in_ and Q_out_ are the inflow and outflow rate (m^3^ min^−1^), and L is the length of the furrow (m), respectively. In the two-point method, the advance curve is determined using Eq. (5)^39,40^.

5 $$x = pt^{r}$$

where *p* and *r* are the fitting parameters, and t is the time from the start of inflow to reach station x. Finally, the other two parameters (*a* and *k*) are determined using Eqs. (6)–(10).

6 $${\upalpha } = \frac{{{\text{log}}\left( {{\raise0.7ex\hbox{${V_{L} }$} \!\mathord{\left/ {\vphantom {{V_{L} } {V_{0.5L} }}}\right.\kern-0pt} \!\lower0.7ex\hbox{${V_{0.5L} }$}}} \right)}}{{{\text{log}}\left( {{\raise0.7ex\hbox{${t_{L} }$} \!\mathord{\left/ {\vphantom {{t_{L} } {t_{0.5L} }}}\right.\kern-0pt} \!\lower0.7ex\hbox{${t_{0.5L} }$}}} \right)}}$$

7 $${\text{k}} = \frac{{V_{L} }}{{\sigma_{Z} t_{L}^{\alpha } }}$$

8 $${\text{V}}_{0.5L} = \frac{{Q_{0} t_{0.5L} }}{{0.5{\text{L}}}} - \sigma_{y} A_{0} - \frac{{f_{0} t_{0.5L} }}{1 + r}$$

9 $${\text{V}}_{L} = \frac{{Q_{0} t_{L} }}{{\text{L}}} - \sigma_{y} A_{0} - \frac{{f_{0} t_{L} }}{1 + r}$$

10 $$\sigma_{z} = \frac{{\alpha + r\left( {1 - \alpha } \right) + 1}}{{\left( {1 + \alpha } \right)\left( {1 + r} \right)}}$$

where σ_y_ is the surface profile shape factor (0.77); σ_Z_ is the subsurface profile shape factor; *A*_0_ is the wetted area at the upstream (m^2^) and t_0.5L_ and t_L_ are the advance times (min) at two points, *x*_1_ = 0.5*L* and *x*_2_ = *L*, respectively.

The two-point method is one of the most practical methods for estimating infiltration parameters in surface irrigation because it is mathematically simple and applicable and requires limited data^41^. For this reason, many researchers have also used this method to determine the infiltration parameters in continuous^13,14,31,42,43^ and surge irrigation^11,28,44^. Ismail and Depeweg^11^ reported that the two-point method has high accuracy in evaluating the contiomus and surge irrigation in short-length furrows. However, the two-point method has also some limitations. The main limitation is the use of two points in field data collection to estimate infiltration parameters. This method has a high sensitivity to advance time measurement^45^. For this purpose, Bautista et al.^41^ recommended increasing the accuracy and measuring more points during the process of data collection. Therefore, in this study, we collected the data at all stations completely and continued the process of data collection until the end of the recession phase to reach the *f*_0_ that would improve the accuracy of the two-point method in estimating the infiltration parameters. Moreover, according to Gillies et al.^46^ using the runoff hydrograph rather than a fixed value of runoff increases the accuracy of the two-point method in estimating infiltration parameters. Therefore, the runoff hydrograph was used to evaluate and simulate the irrigation process.

In surge irrigation, soil wetting can be expressed by two independent equations. However, it seems that neither of the equations can properly consider the second wetting because the wetted perimeter changes significantly between the first and third cycles. The soil wetting process is described by two independent equation in surge irrigation, both of which consider the first and third cycles as the wetted perimeter^47^. The infiltration parameters of *k* and *a* of the Kostiakov-Lewis equation were determined using the two-point method for the first cycle^11^. The *f*_0_ was measured using Eq. (4) when furrows were wetted by the last surge and runoff started^22,45^.

#### Determination of cut-off time

The parameters of the Kostiakov-Lewis infiltration equations (Eq. 3) were first determined in the first set of furrows based on the Elliot and Walker^39^. For this purpose, eight experimental furrows were used to measure the infiltration parameters of the Kostiakov-Lewis equation (a combination of stream size of 0.4 l/s and 0.6 l/s and furrow lengths of 70 m and 90 m under continuous and surge irrigation methods). Table 3 shows the field-measured parameters *a*, *k* and *f*_0_. Then, in the second adjacent set of furrows, the advance-recession times, infiltrated water depth, volume of applied water, and runoff hydrograph were monitored and measured for irrigation performance analyses based on addressing the required irrigation depth of Z_req_ = 0.05 m at the end of the furrows^40,48^. Determination of cutoff time is important for addressing the required infiltrated depth. Cutoff time in every furrow of the second set was calculated according to Eqs. (11) and (12). Due to different cutoff times, the total volume of applied water in each furrow (V_in_, m^3^) was different (Table 2) because T_a_ and T_z_ differed.

11 $$Z_{req} = k T_{Z}^{a} + f_{0} T_{Z}$$

12 $$T_{co} = T_{z} + T_{a}$$

where ${T}_{a}$ is advance time, ${T}_{co}$ is cutoff time, and ${T}_{Z}$ is opportunity time and *a*, *k* and *f*_0_ given Table 3. Since the unit of *Z*_*req*_ is m^3^ m^−1^, the required amount of applied water of 0.05 m was multiplied by the furrow width as 0.75 m.

**Table 3:** Field-measured Kostiakov-Lewis infiltration parameters and the subsurface profile shape factor of the continuous and surge irrigations based on the two-point method.

	Furrow name	*a*	*k*	*f*_0_	$\sigma_{Z}$
Continuous irrigation	Q_1_L_1_	0.25	0.0021	0.00023	0.834
	Q_1_L_2_	0.26	0.0018	0.00022	0.830
	Q_2_L_1_	0.26	0.0035	0.00019	0.839
	Q_2_L_2_	0.25	0.0031	0.00024	0.838
Surge irrigation in the 1st cycle	Q_1_L_1_R_0.5_	0.16	0.0028	0.00022	0.901
	Q_1_L_2_R_0.5_	0.38	0.0016	0.00022	0.724
	Q_2_L_1_R_0.5_	0.49	0.0029	0.00020	0.658
	Q_2_L_2_R_0.5_	0.37	0.0030	0.00024	0.687
Surge irrigation in the 3rd cycle	Q_1_L_1_R_0.5_	0.60	0.0004	0.00022	0.681
	Q_1_L_2_R_0.5_	0.27	0.0018	0.00022	0.818
	Q_2_L_1_R_0.5_	0.36	0.0030	0.00020	0.758
	Q_2_L_2_R_0.5_	0.35	0.0024	0.00024	0.783

Q: stream size; L: furrow length; R_0.5_: surge irrigation with cycle ratio of 0.5.

*k* and *a*: empirical coefficients (*a*:dimensionless, *k*: m^3^ min^−α^ m^−1^); *f*_0_ basic infiltration rate (m^3^m^−1^ min^−1^); σ_z_: subsurface profile shape factor.

**Figure 2:**
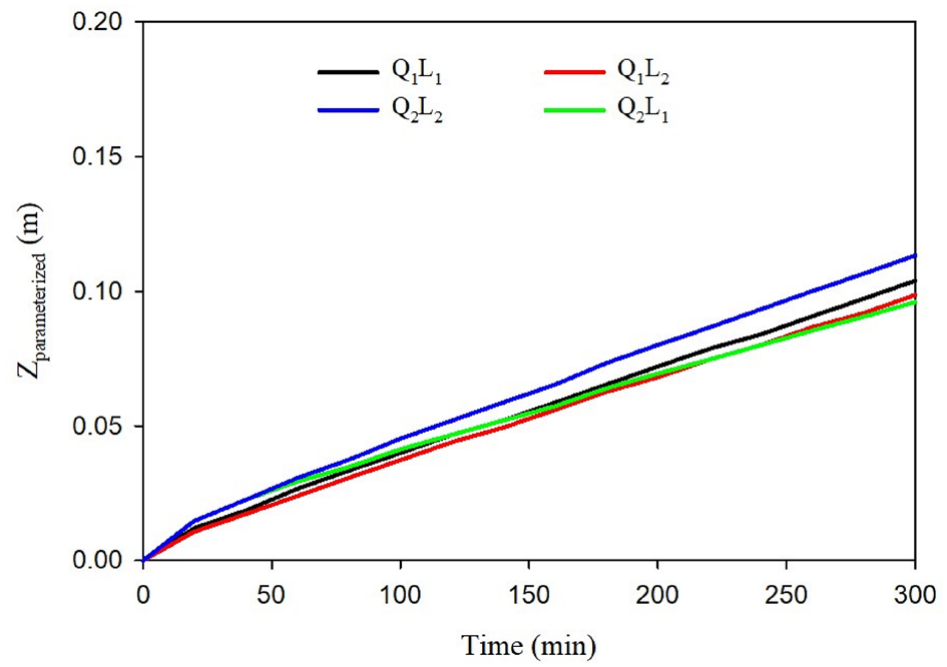
Estimated infiltrated water depth (Z_parameterized_) for the continuous irrigation corresponding to the parameterized Kostiakov-Lewis equation as Table 3.

It is noteworthy that conducting the experiment on two sets of furrows would consider soil spatial variability and its likely effect on the variability of infiltration parameters^49,50^. Although infiltration may change because of spatial variability^51^ and the changes could be even in many orders of magnitude within short distances^52^, former studies on the experimental field showed that the spatial infiltration variability was negligible^53,54^. Nevertheless, because of the time gap between the former researches in 2002 and this research, our further confirmatory field-based analyses showed that the application of the parameterized infiltration equations obtained under a combination of stream size (Q) and furrow length (L) did not reveal high spatial variability in infiltration, and the measurements done in different furrows resulted in nearly similar estimated infiltrated water depth, Z_parametrized_ (Fig. 2). In this regard, Trout and Mackey^55^, after extensive field measurements, reported that the furrow-to-furrow infiltration variability is basically due to uneven tractor and implement wheel compaction of furrows, which was not the case in our small experimental field. In addition, according to a detailed soil survey in the study area^56^, the soil texture of the experimental site is uniform, which also confirms the lack of influential infiltration variability^49^.

### Models’ description and application

#### WinSRFR model

The WinSRFR model is a software package for hydraulic analysis of surface irrigation systems, developed by the Agricultural Research Service of the United States Department of Agriculture^57,58^. WinSRFR is the combination of three earlier models: BORDER^59^, BASIN^60^, and SRFR^61^. The WinSRFR is built based on four analytical functionalities namely; Event Analysis, Simulation, Physical Design, and Operations Analysis. In this study, Event Analysis and Simulation were used to calibrate the model, and then to simulate and evaluate the performance of the continuous and surge irrigations, respectively^58^. The zero-inertia model was used for simulation and evaluation of furrow irrigation in WinSRFR version 5.1^58^ due to open-ended boundary conditions and low slopes in furrows.

According to Ojaghlou et al.^28^, surge irrigation affects the infiltration process differently from continuous irrigation. Because of the effect of the stream size in surge irrigation, the infiltration parameters in each cycle need to be determined separately^28,44^. For this purpose, the concept of Cycle Ratio-Time Model (CRTM) of Blair and Smerdon^47^ has been used in the WinSRFR. The concept of CRTM is that the infiltration rate continues to decrease during the off-time, just as if water was flowing continuously^47^. Hence, the opportunity time at any point is a function of the total time^58^. The option can be practically used with any infiltration formulation. We therefore used the Kostiakov-Lewis infiltration equation for simulating the surge irrigation in WinSRFR.

#### SIRMOD model

The SIRMOD model was first developed at Utah State University in 1987, and its latest version was proposed in 2005^62^. The SIRMOD simulates all surface irrigation methods, including furrow, border, and basin irrigation systems on the field, and helps in the evaluation and simulation of physical design and inflow variables. The most important influential parameters in SIRMOD are basic infiltration rate, stream size, cycle on-time, and cycle ratio^11^. Similar to WinSRFR, the zero-inertia model was used in simulating with SIRMOD.

For simulation of surge irrigation in SIRMOD, the first cycle (as a dry, continuous condition) and the third cycle (as wet, intermittent flow condition) are used to estimate the infiltration parameters by the two-point method as recommended by Ismail and Depeweg^11^, Horst et al.^18^, and Ojaghlou et al.^28^. The resulting equations are:

13 $$Z_{c} = k\tau^{a} + f_{0} \tau$$

14 $$Z_{s} = k^{\prime}\tau^{{a^{\prime}}} + f_{0}^{^{\prime}} \tau$$

where *Z*_*c*_ and *Z*_*s*_ are the infiltrated volumes per unit furrow length (m^3^ m^−1^) for continuous (dry) and intermittent flow (wet) conditions, respectively. The *k*, *k'*, *a*, *a'*, *f*_0_ and *f*_0_*'* are the empirical parameters that depend on the soil type and the effect of cycled wetting and drying, and τ is the cumulative opportunity time over all the surges applied^11^. The infiltration in the second cycle is described by a transition function as described in Walker and Humpherys^63^, Ismail and Depeweg^11^ and Horst et al.^18^.

### Calibration of infiltration parameters using WinSRFR and SIRMOD

The calibration of the infiltration equations by WinSRFR and SIRMOD was done by the trial-and-error^14,64^. To calibrate the continuous irrigation in SIRMOD, the initially field-measured values of the non-conservative parameters *a*, *k*, and *f*_0_ were fine-tuned and calibrated. However, the Manning roughness coefficient (*n* = 0.04) was considered as a conservative parameters without any change. Then by changing the infiltration coefficients in an acceptable range (NRMSE < 10%), the match between the measured and simulated advance-recession times, infiltrated water depth, and runoff were assessed. To calibrate the infiltration parameters in surge irrigation in SIRMOD, the measured infiltration equations from the first and third cycles were calibrated. The values of the calibrated parameters in SIRMOD were determined by trial and error to find the best match between the measured and simulated advance-recession times^18,65^ (Table 4).

**Table 4:** Calibrated values of the non-conservatives infiltration parameters of the continuous and surge irrigations in WinSRFR and SIRMOD.

		Continuous irrigation			Surge irrigation in the 1st cycle			Surge irrigation in the 3rd cycle		
		a_cal_	k_cal_	f_0cal_	a_cal_	k_cal_	f_0cal_	a_cal_	k_cal_	f_0cal_
WinSRFR	Q_1_L_1_	0.23	0.0038	0.00021	0.10	0.0086	0.00022	–	–	–
	Q_1_L_2_	0.24	0.0024	0.00022	0.15	0.0049	0.00021	–	–	–
	Q_2_L_1_	0.24	0.0048	0.00019	0.48	0.0030	0.00018	–	–	–
	Q_2_L_2_	0.24	0.0029	0.00025	0.25	0.0040	0.00021	–	–	–
SIRMOD	Q_1_L_1_	0.27	0.0026	0.00020	0.48	0.0019	0.00022	0.45	0.0023	0.00011
	Q_1_L_2_	0.26	0.0017	0.00021	0.50	0.0015	0.00021	0.36	0.0016	0.00008
	Q_2_L_1_	0.28	0.0029	0.00019	0.45	0.0021	0.00025	0.23	0.0025	0.00015
	Q_2_L_2_	0.21	0.0024	0.00023	0.48	0.0019	0.00022	0.45	0.0009	0.00011

Q: stream size; L: furrow length; R_0.5_: surge irrigation with cycle ratio of 0.5.

*k* and *a*: empirical coefficients (*a*:dimensionless, *k*: m^3^ min^−α^ m^−1^); *f*_0_ basic infiltration rate (m^3^m^−1^ min^−1^).

To calibrate the non-conservative infiltration parameters for the continuous irrigation in the WinSRFR, Merriam and Keller^66^ method was chosen under the Event Analysis world option^14,43^. In this regard, the field-measured values of *a* and *f*_0_ were set as non-conservative infiltration parameters. Then by changing *a* and *f*_0_ the match between the measured and simulated advance-recession times, infiltrated water depth, and runoff was assessed (NRMSE < 10%), and if the match values were poor, then new values of *a* and *f*_0_ would be tested.

Because of the inability of the Event Analysis world of WinSRFR to evaluate surge irrigation, the Simulation world was used to calibrate the infiltration parameters of surge irrigation. Besides, as the Merriam-Keller method uses runoff in the simulation and there is no runoff in the first surge under surge irrigation, the Merriam-Keller method cannot be used to calibrate the infiltration parameters of surge irrigation. It should also be noted that to calibrate the infiltration parameters of surge irrigation in the Simulation world, the infiltration parameters *a* and *k* of the first surge and final *f*_0_ were used. The values of the calibrated parameters in WinSRFR were determined by trial and error to find the best match between the measured and simulated advance-recession times and infiltrated water depth (Table 4).

### Irrigation performance indices

Four irrigation performance indices were used to analyze the irrigation performance including application efficiency (*AE*), distribution uniformity (*DU*), deep percolation (*DP*), and tail-water ratio (*TWR*)^12^. It is noteworthy that irrigation method performance is generally assessed using the distribution uniformity index, while the irrigation management performance is assessed with the application efficiency or the fraction of beneficial water use^4^.

15 $$AE = \frac{{D_{ad} }}{{D_{ap} }} \times 100$$

16 $$DU = \frac{{D_{min} }}{{D_{avg} }} \times 100$$

17 $$DP = \frac{{D_{dp} }}{{D_{ap} }} \times 100$$

18 $$TWR = \frac{{D_{ro} }}{{D_{ap} }} \times 100 = \left( {{1}00 - AE - DP} \right)$$

where *D*_*ad*_, *D*_*ap*_, *D*_*min*_, *D*_*avg*_, *D*_*dp*_, and *D*_*ro*_ are the depth of total water stored in the 1 m soil profile over the furrow length (mm); depth of water applied to the furrow (mm); minimum depth of infiltrated water (mm); the average depth of infiltrated water over the furrow length (mm), depth of deep percolated water (mm), and the depth of runoff (mm), respectively.

### Evaluation of irrigation models

In order to evaluate the performance of the SIRMOD and WinSRFR models in simulating the observed field data of advance and recession times, and infiltrated water depths, the Normalized Root Mean Squared Error (NRMSE)^67^, the refined Willmott Index of Agreement (dr)^68^, and the Nash–Sutcliffe efficiency coefficient (NS)^69^ which is also known as Model Efficiency were used. The runoff volume was assessed by the Relative Error (RE) because we had only one observed value of the total runoff volume and one value for the simulated runoff volume by each model. Accordingly, these indices are calculated by the following equations.

19 $$NRMSE = \frac{1}{{\overline{O} }} \times \sqrt {\frac{{\sum\limits_{i = 1}^{N} {(O_{i} - P_{i} )^{2} } }}{N}}$$

20 $$d_{r} = \left\{ \begin{gathered} 1 - \frac{{\sum\limits_{i = 1}^{n} {\left| {P_{i} - O_{i} } \right|} }}{{c\sum\limits_{i = 1}^{n} {\left| {O_{i} - \overline{O} } \right|} }} \hfill \\ When,\sum\limits_{i = 1}^{n} {\left| {P_{i} - O_{i} } \right|} \le c\sum\limits_{i = 1}^{n} {\left| {O_{i} - \overline{O} } \right|} \hfill \\ 1 - \frac{{c\sum\limits_{i = 1}^{n} {\left| {O_{i} - \overline{O} } \right|} }}{{\sum\limits_{i = 1}^{n} {\left| {P_{i} - O_{i} } \right|} }} \hfill \\ When,\sum\limits_{i = 1}^{n} {\left| {P_{i} - O_{i} } \right|} > c\sum\limits_{i = 1}^{n} {\left| {O_{i} - \overline{O} } \right|} \hfill \\ \end{gathered} \right.$$

21 $$NS = 1 - \left[ {\frac{{\sum\limits_{i = 1}^{N} {\left( {O_{i} - P_{i} } \right)^{2} } }}{{\sum\limits_{i = 1}^{N} {\left( {O_{i} - \overline{O} } \right)^{2} } }}} \right]$$

22 $$RE = \frac{{P_{i} - O_{i} }}{{O_{i} }} \times 100$$

where O_i_ and P_i_ are the observed and simulated values, N is the number of observations and the $\overline{\mathrm{O} }$ is the average observation value (c is equal to 2).

NRMSE indicates the simulation error (%). NRMSE is very useful when large simulation errors are unfavorable^70^. The simulation is excellent if NRMSE is less than 10%, good if NRMSE is greater than 10% and less than 20%, fair if NRMSE is greater than 20% and less than 30%, and poor if NRMSE is greater than 30%. d_r_ is proposed to measure the degree that the observed data are approached by the simulated data. It ranges from − 1 to 1, and − 1 shows no agreement, and 1 indicates a perfect match between the simulated and observed data^68^. The NS varies from − ∞ to 1, with higher values showing better agreement. If the value of NS is negative, the model simulation is worse than the average of observations^71,72^. RE is used to determine the error percentage of simulation in comparison to field measurement. RE is positive or negative according to whether the simulated values are an overestimate or an underestimate compared to observed values^73^. It is worth mentioning that the statistics of the model evaluation are affected by large errors and values which are extreme, especially in the case of small datasets. This mainly happens because of squared differences^74^. But, it should be mentioned that these parameters are all subjective because there are a large number of data points, repeated data, and the existence of outliers. Besides, a set of the indices should be used to be able to interpret the model performance^74^.

## Results

### Advance and recession times

#### Continuous irrigation

**Figure 3:**
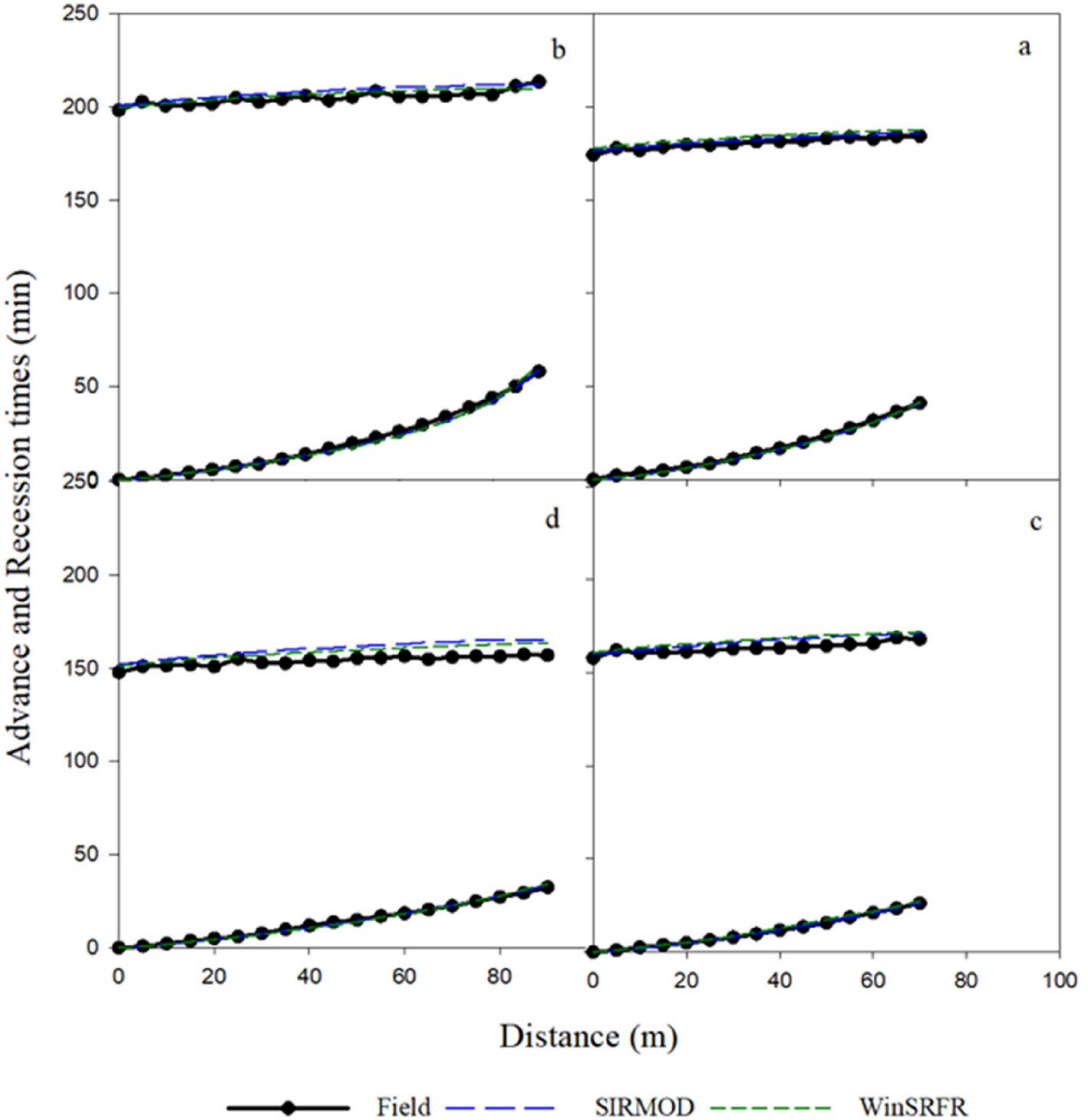
Comparison of the measured and simulated advance-recession times under continuous irrigation (**a**. Q_1_L_1_, **b**. Q_1_L_2_, **c**. Q_2_L_1_ and **d**. Q_2_L_2_).

The comparisons of the observed and simulated advance-recession times under continuous irrigation are shown in Fig. 3. The evaluation of the advance-recession times by the SIRMOD and WinSRFR models based on statistical indicators are presented in Table 5. The highest values of NRMSE for the simulation of advance time by SIRMOD and WinSRFR were 4.31 and 7.68%, and the highest values of NRMSE in simulating recession time were 4.30 and 3.13% for SIRMOD and WinSRFR, respectively. These results indicate the excellent accuracy of both models in simulating advance-regression times under continuous irrigation (the values of NRMSE in all treatments are less than 10%).

**Table 5:** Evaluation of the advance-recession times by the SIRMOD and WinSRFR models under the continuous and surge irrigations based on the statistical indicators.

		Length (m)	Inflow (l/s)	Advance time			Recession time		
				NRMSE (%)	NS (-)	dr (-)	NRMSE (%)	NS (-)	dr (-)
SIRMOD	Q_1_L_1_	70	0.4	4.31	0.99	0.97	0.91	0.67	0.67
	Q_1_L_2_	90	0.4	3.83	0.99	0.97	1.83	0.77	0.37
	Q_2_L_1_	70	0.6	2.54	0.99	0.98	2.09	− 0.54	0.25
	Q_2_L_2_	90	0.6	2.68	0.99	0.97	4.30	− 5.42	0.33
	Q_1_L_1_R_0.5_	70	0.4	1.66	0.92	0.85	6.12	0.97	0.73
	Q_1_L_2_R_0.5_	90	0.4	2.23	0.56	0.68	4.43	0.98	0.72
	Q_2_L_1_R_0.5_	70	0.6	3.05	0.91	0.89	9.00	0.94	0.60
	Q_2_L_2_R_0.5_	90	0.6	2.87	0.68	0.73	6.07	0.95	0.60
WinSRFR	Q_1_L_1_	70	0.4	3.04	0.99	0.97	1.29	− 0.25	0.35
	Q_1_L_2_	90	0.4	7.68	0.99	0.96	1.74	0.30	0.70
	Q_2_L_1_	70	0.6	7.49	0.99	0.95	2.63	− 1.44	0.05
	Q_2_L_2_	90	0.6	6.39	0.99	0.96	3.13	− 2.30	0.07
	Q_1_L_1_R_0.5_	70	0.4	5.26	0.16	0.97	5.29	0.96	0.71
	Q_1_L_2_R_0.5_	90	0.4	3.43	1.00	0.19	2.07	0.97	0.44
	Q_2_L_1_R_0.5_	70	0.6	3.42	0.89	0.85	8.15	0.93	0.59
	Q_2_L_2_R_0.5_	90	0.6	4.85	0.11	0.79	5.29	0.94	0.53

Q: stream size; L: furrow length; R_0.5_: surge irrigation with cycle ratio of 0.5.

Except for the Q_2_L_2_ which simulated with SIRMOD, Table 5 shows that the simulations of recession times in SIRMOD and WinSRFR were more accurate than the simulations of advance time under continuous irrigation (mean values of NRMSE in SIRMOD and WinSRFR for recession time were 2.28 and 2.20%, respectively, while they were 3.32 and 6.15% for advance time, respectively). Averaged over the furrow length, increasing the stream size from 0.4 l/s to 0.6 l/s slightly reduced the accuracy of WinSRFR (leading to an increase in NRMSE from 5.36 to 6.94%) while increased the accuracy of SIRMOD (NRMSE decreased from 4.07 to 2.61%) in the simulation of advance time under continuous irrigation (Fig. 3 and Table 5). However, the simulation accuracy of the advance-recession times in SIRMOD and WinSRFR was higher in furrow length of 70 m (L_1_) than 90 m (L_2_). These results indicate that the furrow length had the greatest effect on the accuracy of SIRMOD, while WinSRFR was influenced by the stream size and cut-off time.

#### Surge irrigation

**Figure 4:**
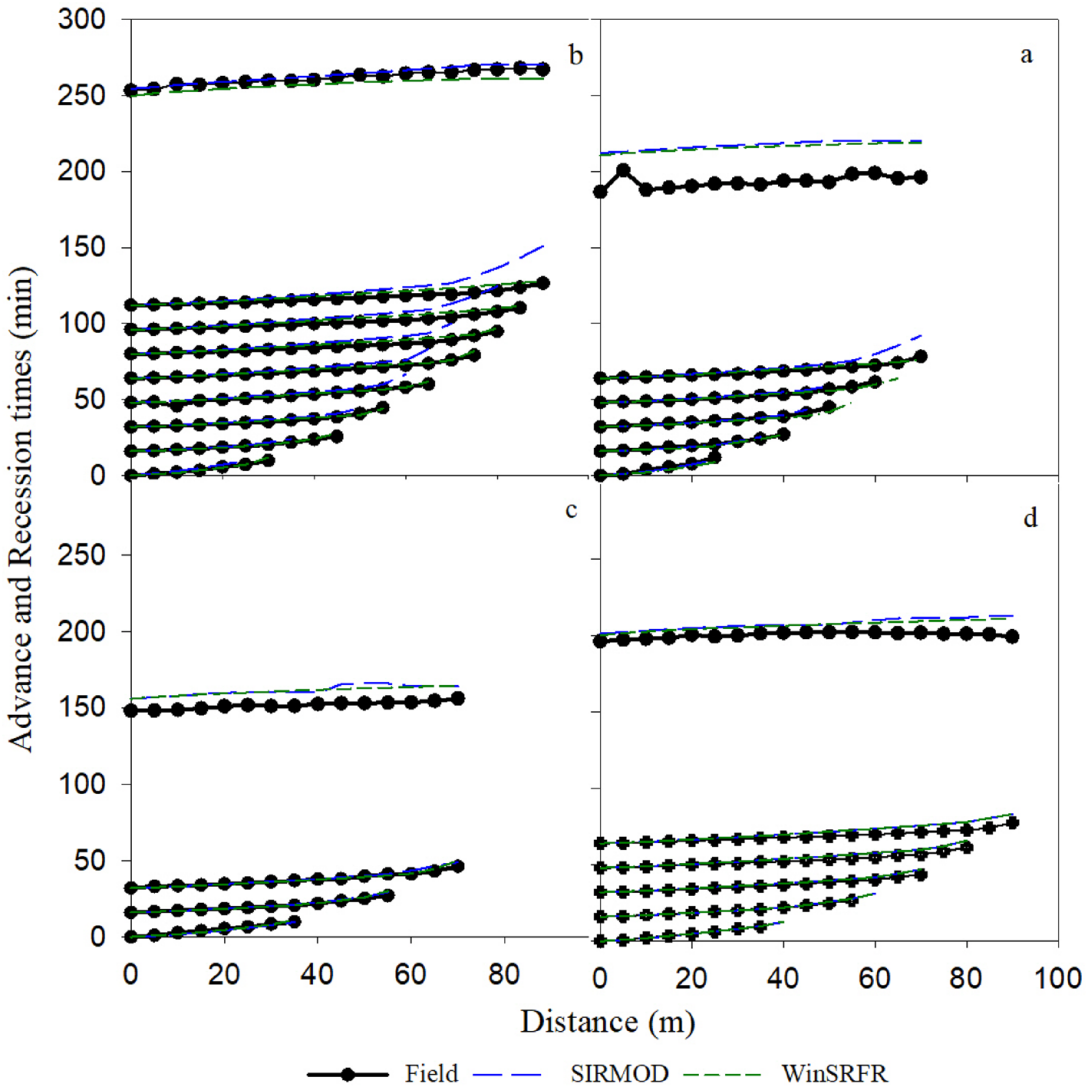
Comparison of the measured and simulated advance-recession times under surge irrigation (**a**. Q_1_L_1_R_0.5_, **b**. Q_1_L_2_R_0.5_, **c**. Q_2_L_1_R_0.5_ and **d**. Q_2_L_2_R_0.5_).

The simulated output of advance and recession times under surge irrigation are presented in Fig. 4 and Table 5. The results revealed that WinSRFR and SIRMOD simulated the advance and recession times excellently (NRMSE < 10%). Figure 4 indicated that the simulation accuracy of recession times under WinSRFR and SIRMOD increases by reducing the number of cycles. For example, Q_2_L_1_R_0.5_ (three cycles) and Q_1_L_2_R_0.5_ (eight cycles) had high and low accuracies in the simulation of recession times. The statistical indicators in Table 5 confirm these results, too. Thus, the simulation of recession time in the Q_2_L_1_R_0.5_ under SIRMOD and WinSRFR was better than the other furrows. Table 5 shows that unlike the continuous irrigation, SIRMOD and WinSRFR fared well in simulating the advance time compared to the recession time in surge irrigation. As seen in Table 5, the value of NRMSE in advance time varied from 1.66 to 3.05% in the SIRMOD and from 3.42 to 5.26% in the WinSRFR; however, the NRMSE values of recession time varied from 4.43 to 9.00% in SIRMOD and from 2.07 to 8.15% in WinSRFR. Overall, the advance-recession times under surge irrigation were simulated with higher accuracy by SIRMOD and WinSRFR (the values of NRMSE in all treatments are less than 10%) (Table 5). Moreover, WinSRFR and SIRMOD simulated the advance-recession times more accurately at the stream size of 0.4 l/s compared to 0.6 l/s (see Fig. 4 and Table 5). Results demonstrated that the accuracy of the SIRMOD model in simulation of the advance-recession times increased with decreasing stream size (Table 5). In addition, changing furrow length from 70 m to 90 m improved the simulation of advance-recession times with SIRMOD and WinSRFR.

### Runoff

**Figure 5:**
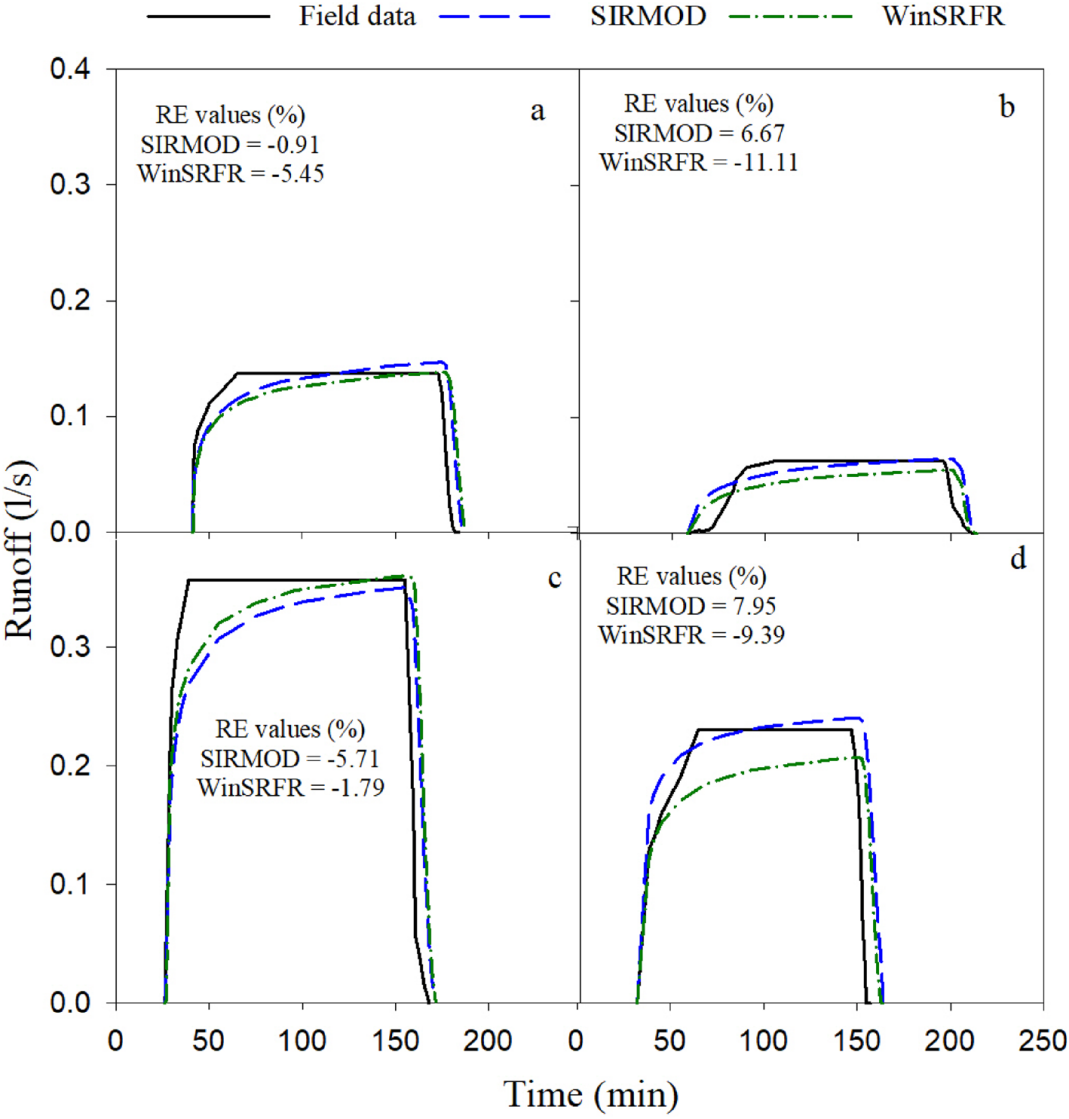
Comparison of the measured and simulated runoff under continuous irrigation (**a**. Q_1_L_1_, **b**. Q_1_L_2_ **c**. Q_2_L_1_ and **d**. Q_2_L_2_).

**Figure 6:**
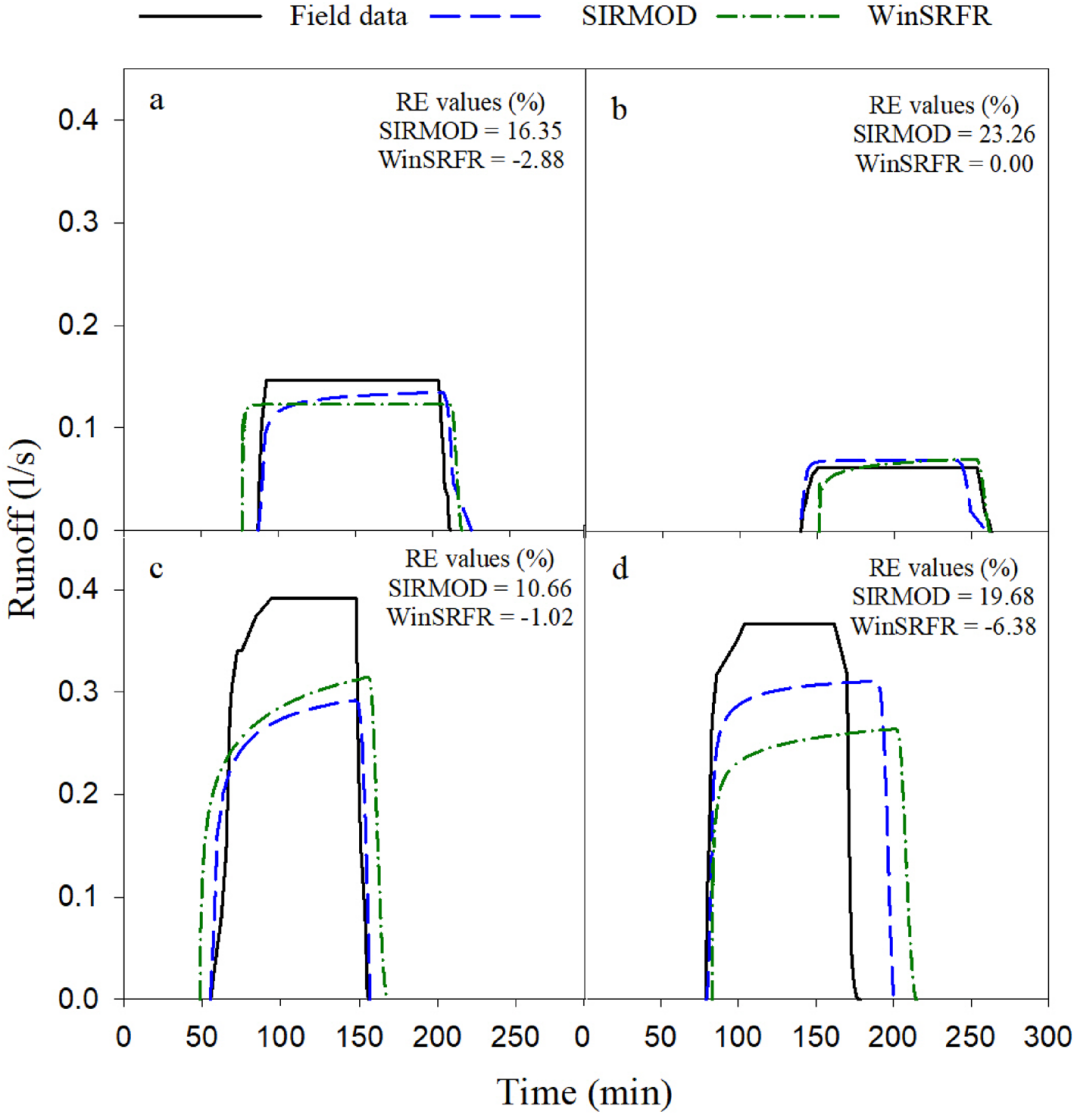
Comparison of the measured and simulated runoff under surge irrigation (**a**. Q_1_L_1_R_0.5_, **b**. Q_1_L_2_R_0.5_, **c**. Q_2_L_1_R_0.5_ and **d**. Q_2_L_2_R_0.5_).

Figures 5 and 6 illustrate the simulation of runoff under continuous and surge irrigation, respectively. To address the required water depth (Z_req_ = 0.05 m) at the end of the furrow in surge irrigation, the inflow was kept running until reaching the cutoff time (T_co_). Therefore, the runoff in the surge irrigation is because of this irrigation management.

#### Continuous irrigation

The comparison of the simulated runoff volumes with field-measured values showed that SIRMOD and WinSRFR models simulated satisfactorily the runoff volumes under continuous irrigation (Fig. 5). Table 2 and Fig. 5 showed that there is a direct relationship between the stream size and the runoff volume such that by increasing the stream size, the runoff volume increased. For instance, Q_2_L_1_ with a stream size of 0.6 l/s and a length of 70 m has the greatest amount of runoff volume. Averaging over furrow lengths indicates that increasing the stream size from 0.4 l/s to 0.6 l/s decreased the accuracy of the SIRMOD model (RE changes from 3.79 to 6.83%) while increased in the accuracy of the WinSRFR model (RE changes from 8.28 to 5.59%) in runoff volume simulation under continuous irrigation. In addition, increasing the furrow length from 70 m to 90 m decreased the accuracy of SIRMOD (RE changes from 3.31 to 7.31%) and WinSRFR (RE changes from 3.62% to 10.25%) in runoff volume simulation such that both models simulated the runoff volume in 70 m long furrows with a small difference compared to the measured runoff volume (Fig. 5). It is noteworthy that WinSRFR underestimated the runoff volume in all combinations of furrow length and stream size, but SIRMOD showed a contrasting behavior such that it overestimated runoff volume in long furrow length (RE > 0 in Fig. 5).

#### Surge irrigation

Simulated runoff in different furrows under surge irrigation is presented in Fig. 6. Since the total volume of applied water was the same for both surge and continuous irrigation, the performance of the models were assessed with runoff percentage that is the ratio of the total runoff volume to the total volume of applied water. The results confirmed that surge irrigation reduced the runoff volume although this amount was small (Table 2 and Fig. 6).

The SIRMOD model could not simulate surge irrigation properly such that in all conditions overestimated the runoff volume (the value of RE varied from 10.66 to 23.26%, in Fig. 6). The results showed that the simulation accuracy of the SIRMOD model was reduced while the WinSRFR model simulated the runoff volume with excellent accuracy though it slightly underestimated the runoff volume (RE < 0 in Fig. 6). It was found that increasing the stream size from 0.4 l/s to 0.6 l/s increased the accuracy of SIRMOD but it was not consistent for WinSRFR (Fig. 6). Likewise, increasing the furrow length from 70 m to 90 m decreased the accuracy of SIRMOD but it was not consistent for WinSRFR models in simulating runoff volume. This shows that models need to be verified under different field managements such as soil type for achieving precise irrigation strategy.

### Infiltrated water depth

#### Continuous irrigation

**Figure 7:**
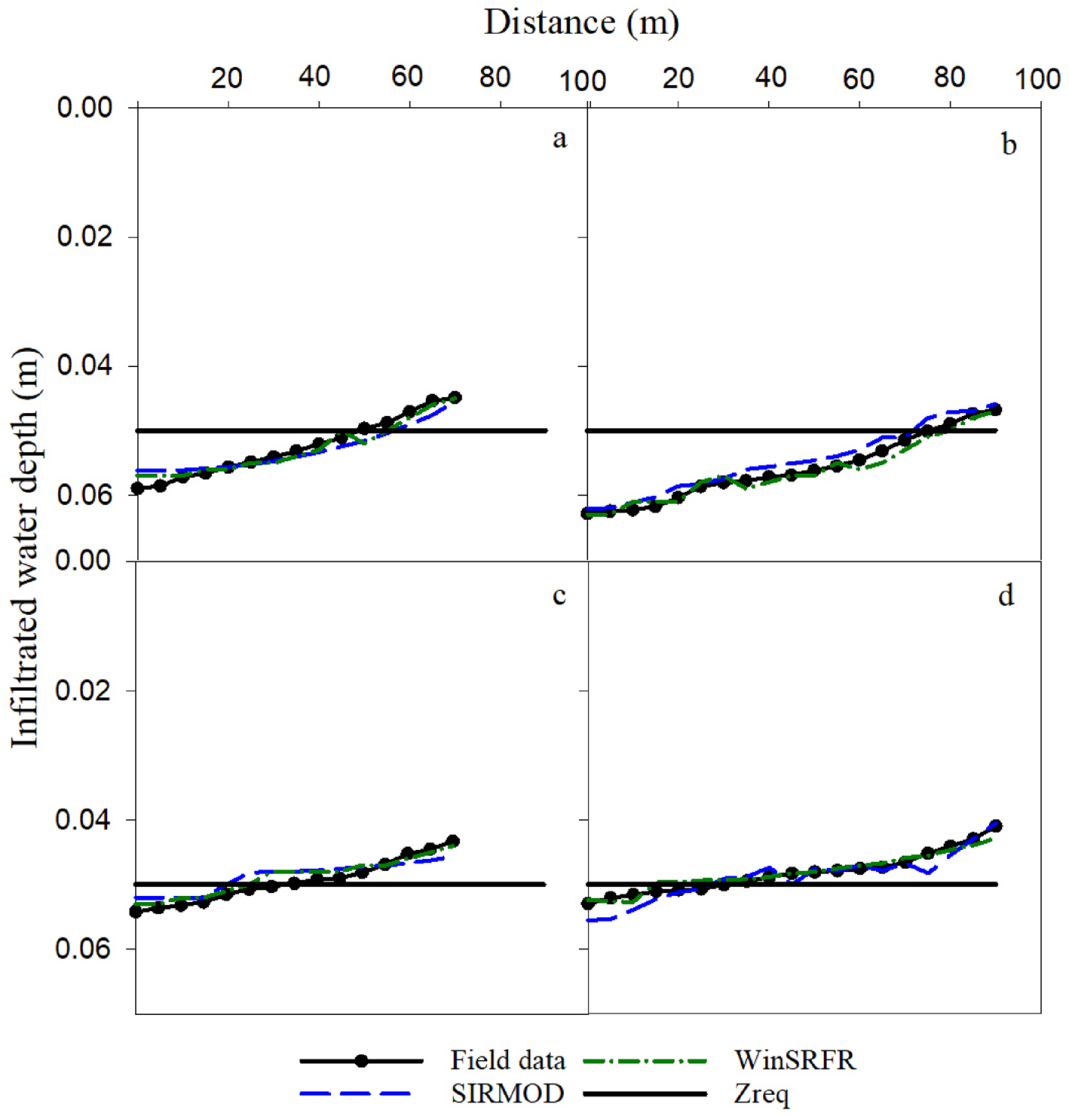
Comparison of the measured and simulated infiltrated depth under continuous irrigation (**a**. Q_1_L_1_, **b**. Q_1_L_2_ **c**. Q_2_L_1_and **d**. Q_2_L_2_).

Table 6 summarizes the statistical indicators regarding the simulation of the infiltrated water depth under continuous irrigation by SIRMOD and WinSRFR models. Based on the NRMSE and the other statistical indicators (d_r_ and NS) in Table 6, WinSRFR and SIRMOD show an excellent fit of the field-measured values and simulated values of the infiltrated water depth (NRMSE < 10%). Increasing the stream size from 0.4 l/s to 0.6 l/s did not have a significant effect on the performance of SIRMOD and WinSRFR models in simulating the infiltration depth under continuous irrigation. Besides, increasing the furrow length from 70 m to 90 m under the stream size of 0.4 l/s decreased the accuracy of the SIRMOD and WinSRFR models. In contrary, increasing the furrow length from 70 m to 90 m under 0.6 l/s increased the simulation accuracy of both models. Table 6 and Fig. 7 show that the uniformity of infiltrated water depth was higher in Q_1_L_1_ and Q_1_L_2_ because of a smaller stream size (0.4 l/s) and greater opportunity time, which led to reaching the required irrigation water depth. However, increasing the stream size from 0.4 l/s to 0.6 l/s under 90 m furrow length resulted in deficit irrigation (i.e., infiltration smaller than required amount 50 mm) in the downstream of furrows (Fig. 7). Overall, the results implied that a precise combination of stream size and furrow length is important in simulating accurate infiltrated water depth.

**Table 6:** Evaluation of the infiltrated water depth by the SIRMOD and WinSRFR models in continuous and surge irrigations based on statistical indicators.

	SIRMOD			WinSRFR		
	NRMSE (%)	NS (-)	dr (-)	NRMSE (%)	NS (-)	dr (-)
Q_1_L_1_	3.09	0.83	0.85	2.75	0.86	0.83
Q_1_L_2_	3.18	0.65	0.85	3.90	0.47	0.76
Q_2_L_1_	3.51	0.80	0.90	3.90	0.76	1.00
Q_2_L_2_	2.13	0.77	0.80	2.93	0.56	0.77
Q_1_L_1_R_0.5_	5.88	0.64	0.60	3.13	0.86	0.60
Q_1_L_2_R_0.5_	4.05	0.69	0.90	1.60	0.95	0.27
Q_2_L_1_R_0.5_	4.22	0.92	0.95	11.77	0.36	0.90
Q_2_L_2_R_0.5_	6.92	0.86	0.86	9.03	− 0.17	0.38

Q: stream size; L: furrow length; R_0.5_: surge irrigation with cycle ratio of 0.5.

#### Surge irrigation

**Figure 8:**
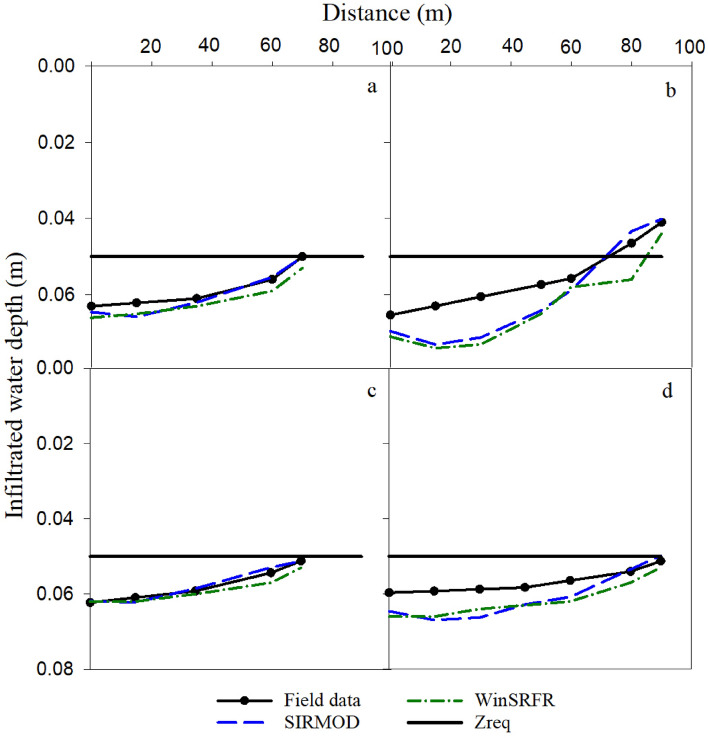
Comparison of the measured and simulated infiltrated depth under surge irrigation (**a**. Q_1_L_1_R_0.5_, **b**. Q_1_L_2_R_0.5_, **c**. Q_2_L_1_R_0.5_ and **d**. Q_2_L_2_R_0.5_).

Infiltrated water depth measured in the surge irrigation and simulated by SIRMOD and WinSRFR under surge irrigation are illustrated in Fig. 8. Comparing Fig. 7 with Fig. 8, it is implied that surge irrigation compared to continuous irrigation resulted in a higher uniformity of infiltrated water depth and prevented the occurrence of deficit irrigation at the end of the furrow. Table 6 shows that SIRMOD simulated the infiltrated water depth successfully under any combinations of stream sizes and furrow length under surge irrigation (NRMSE = 4.05% − 6.92%). However, WinSRFR had much better simulation of the infiltered water depth under surge irrigation by reducing the stream size from 0.6 l/s (NRMSE = 11.77% and 9.03%) to 0.4 l/s (NRMSE = 3.13% and 1.60%) for a fixed furrow length. These findings reveal that while WinSRFR performs better for simulating infiltered water depth under smaller rates of stream sizes, SIRMOD performed successfully under both low and high stream sizes. The least amount of NRMSE was obtained in Q_1_L_2_R_0.5_ for both SIRMOD and WinSRFR. In general, the results of the SIRMOD and WinSRFR models indicated that longer furrows (90 m) resulted in more accurate simulation than shorter furrows (70 m) (Table 6), which could be due to the higher number of cycles and more uniform infiltration.

### Irrigation performance indicators

#### Measured irrigation performance indices

The irrigation performance indices measured in the field and simulated by WinSRFR and SIRMOD under continuous and surge irrigation are summarized in Table 7. The results show that surge irrigation improved irrigation performance indices compared to continuous irrigation in the field (Table 7). In general, continuous irrigation resulted in higher water loss i.e. *DP* and *TWR* than surage irrigation. This caused lower irrigation performance in continuous irrigation compared with surge irrigation Surge irrigation increased the *AE* and *DU* (except in Q_2_L_1_R_0.5_ that reduced *DU* by 3.14%). These results indicate a potential of obtaining greater irrigation performances of surge irrigation compared to continuous irrigation for on-farm water management.

**Table 7:** Irrigation performance indices (%) measured in the field and simulated by SIRMOD and WinSRFR for the continuous and surge irrigations.

	Performance indicators	Continuous irrigation				Surge irrigation			
		Q_1_L_1_	Q_1_L_2_	Q_2_L_1_	Q_2_L_2_	Q_1_L_1_R_0.5_	Q_1_L_2_R_0.5_	Q_2_L_1_R_0.5_	Q_2_L_2_R_0.5_
Field data	AE	63.00	72.00	47.00	64.00	74.80	83.57	65.00	64.75
	DU	89.00	86.00	95.00	91.00	92.51	98.83	91.86	93.18
	DP	11.00	18.00	9.00	9.00	10.75	10.38	6.00	6.46
	TWR	26.00	10.00	44.00	27.00	14.45	6.05	29.00	28.79
SIRMOD	AE	63.00 ***(0.00)***	71.00 ***(***− ***1)***	47.00 ***(0.00)***	62.00 ***(− 2)***	74.35 ***(− 0.45)***	76.59 ***(− 6.98)***	85.28 ***(20.28)***	72.20 ***(7.45)***
	DU	95.00 ***(6)***	95.00 ***(9)***	97.00 ***(2)***	96.00 ***(5)***	78.90 ***(− 13.61)***	85.74 ***(− 13.09)***	65.24 ***(− 26.62)***	78.52 ***(− 14.66)***
	DP	11.00 ***(0.00)***	18.00 ***(0.00)***	8.00 ***(− 1)***	8.00 ***(− 1)***	10.33 ***(− 0.42)***	11.74 ***(1.36)***	11.77 ***(5.77)***	5.64 ***(− 0.82)***
	TWR	26.00 ***(0.00)***	11.00 ***(1)***	45.00 ***(1)***	30.00 ***(3)***	15.32 ***(0.87)***	11.67 ***(5.62)***	2.95 ***(− 26.05)***	22.16 ***(− 6.63)***
WinSRFR	AE	63.00 ***(0.00)***	72.00 ***(0.00)***	47.00 ***(0.00)***	64.00 ***(0.00)***	67.00 ***(− 7.8)***	78.00 ***(− 5.57)***	57.00 ***(− 8)***	61.00 ***(− 3.75)***
	DU	89.00 ***(0.00)***	81.00 ***(− 5)***	94.00 ***(− 1)***	90.00 ***(− 1)***	80.00 ***(− 12.51)***	65.00 ***(− 33.83)***	88.00 ***(− 3.86)***	76.00 ***(− 17.18)***
	DP	12.00 ***(1)***	20.00 ***(2)***	7.00 ***(− 2)***	10.00 ***(1)***	13.00 ***(2.25)***	15.00 ***(4.62)***	5.00 ***(− 1)***	9.00 ***(3.54)***
	TWR	25.00 ***(− 1)***	8.00 ***(− 2)***	46.00 ***(2)***	26.00 ***(− 1)***	20.00 ***(5.55)***	7.00 ***(0.95)***	38.00 ***(9)***	30.00 ***(1.21)***

Q: stream size; L: furrow length; R_0.5_: surge irrigation with cycle ratio of 0.5.

The *Italic* values in parenthesis (%) are the difference between the simulated value and the corresponding measured value.

The comparison of different stream sizes indicated that increasing the stream size from 0.4 l/s to 0.6 l/s decreased *AE* by 12.00 and 14.31%, and *DP* by 5.50 and 4.34%, and increased *TWR* by 17.50 and 18.65%, on average, in both continuous and surge methods, respectively. However, changing the stream size increased *DU* by 5.50% in continuous irrigation and decreased it by 3.15% in surge irrigation method (Table 7). Moreover, comparing furrow lengths of 70 m and 90 m had higher impact on increasing *AE* and *TWR* under continuous than surge irrigation improved *AE* in continuous. Additionally, on average, increasing the furrow length reduced *DU* by 3.50% in continuous irrigation and increased it by 3.82% in surge irrigation. However, increasing furrow length did not affect *DP* remarkably in both continuous and surge methods, except in continuous irrigation that *DP* increased in longer furrow under 0.4 l/s (Table 7).

#### Simulated irrigation performance indices with SIRMOD

The results of irrigation performance indices by SIRMOD under continuous and surge irrigation are summarized in Table 7. The results showed that SIRMOD simulated continuous irrigation with high accuracy. SIRMOD model had excellent accuracy in simulating all irrigation performance indiecs under continuous and surge irrigation, except for the *DU* under surge irrigation that underestimated between 13.09 and 26.62%. This reveals that SIRMOD does not simulate distribution uniformity as well as the other irrigation indices; yet it is functional, useful, and productive in simulating *DU* for scenario managements.

Comparing the stream sizes showed that under a constant furrow length, the lower stream size of 0.4 l/s increased *DP* but decreased *TWR* under continuous irrigation compared to the higher stream size of 0.6 l/s (Table 7). Besides, increasing the stream size from 0.4 l/s to 0.6 l/s in surge irrigation consistently increased *AE* but decreased reduced *DU* under a constant furrow length (Table 7). However, the effects of increasing stream size on *DP* and *TWR* were not consistent under the furrow lengths in surge irrigation. It showed that, although increasing the stream size reduced *DP* from 11.74 to 5.64% under 90 m furrow length, it slightly increased *DP* at 70 m from 10.33 to 11.74%. However, a vice versa result was found for *TWR* under surgae irrigation.

Further analysis showed that the simulated *TWR* consistently decreased by changing the furrow length from 70 m to 90 m under continuous irrigation, which instead increased considerably the simulated *AE*. Moreover, the results of surge irrigation showed that by increasing the furrow length from 70 m to 90 m under lower stream size of 0.4 l/s, *AE and DU* increased by 2.24 and 6.84%, respectively, and *TWR* decreased by 3.65%. However, at higher stream size of 0.6 l/s, increasing the furrow length reduced *AE* and *DP* by 13.08% and 6.13%, respectively, but increased *DU* and *TWR* by 13.28 and 19.21%. These contrasting effects of stream size and furrow length on the irrigation performance indices under surage and contnoius irrigation reveals a complex systems that modelling can help in defining the best irrigation syetsm for a region.

#### Simulated irrigation performance indices with WinSRFR

The simulations of irrigation performance indices of the continuous and surge irrigation by WinSRFR are summarized in Table 7. The WinSRFR showed a high accuracy in simulating the continuous and surge irrigations in compared to the field data, although simulations were slightly better under continuous than surge irrigation. The results demonstrated that WinSRFR simulated all irrigation performance indices under continuous and surge irrigation excellently, except for *DU* under surge irrigation that was underestimated between 3.86 and 33.83%. This reveals that WinSRFR does not simulate distribution uniformity as well as the other irrigation indices except under *Q*_*2*_*L*_*1*_*R*_*0.5*_ that *DU* was simulated well. However, the simulations of *DU* are still good and acceptable. Increasing the furrow length from 70 m to 90 m, and increasing stream size from 0.4 l/s to 0.6 l/s (except under 70 m) consistently increased *AE* and *DP* and decreased *DU* and *TWR* under surge irrigation (Table 7). Overall, the results showed that WinSRFR was more accurate in continuous irrigation than in surge irrigation.

## Discussion

Runoff volume is an important parameter in evaluating the surface irrigation systems. Surge irrigation produced less runoff volume (Fig. 6) and this is an important advantage of using surge irrigation^12,18^. There are several reasons that affect infiltration process in surge irrigation. Expansion of clay particles, reduction of soil hydraulic gradient as soil becomes wet, consolidation of surface soil layer during the off-time, hysteretic behavior of the soil hydraulic properties, air entry in to the soil and entrapment that occurs between surges, and surface sealing due to clogging from sediment particles are the main factors that may reduce infiltration in surge irrigation compared to continuous irrigation^47,75^.

In surge irrigation, since water enters the field during different on-time cycles, the opportunity time would increase that would result in more uniform infiltration^23,27^. This results in a higher proportion of the volume of applied water infiltrates into the soil during the whole irrigation event, which would ultimately reduce runoff volume^76,77^. Our results showed that increasing the furrow length and decreasing the stream size reduced the runoff volume because under a longer furrow length and lower stream size, it takes a longer time for completing the advance phase, which leads to a decrease in the runoff volume. Other researchers reported that in a clay soil, stream size should increase with longer furrow lengths in order to obtain high application efficiencies^78^. These inconsistencies reveal that soil type and spatial variability are critical in furrow management^51,79^. Thus, surge irrigation with the optimum combination of stream size and furrow length may reduce runoff volume and subsequently improve the precise irrigation in areas with limited water availability.

Interestingly, surge irrigation significantly eliminated deficit irrigation at the lower sections of the furrows (Figs. 7 and 8). This is a major advantage of surge irrigation compared to continuous irrigation because in those areas that there are facing lack of water for irrigation, adapting surge irrigation to eliminate or reduce deficit irrigation at the lower part of the furrow could be regarded as a climate-smart irrigation management. In fact, because of the higher soil moisture in the first cycles, the water front moves more rapidly to the end of the furrow in the next cycles. As a result, required water was reached at the lower stations, which reduces water losses as deep percolation in the upper stations^11,35,80^.

### Continous and surge irrigation performances

One of the main objectives of this study was to investigate irrigation performance indicators under surge irrigation compared with continuous irrigation that has not been sufficiently studied by other researchers. Overall, surge irrigation decreased deep percolation and runoff and in turn increased the application efficiency and distribution uniformity (Table 7), which are the favoured reasons for practicing surge irrigation. In line with our argument, Kifle et al.^12^ also showed that surge irrigation improved irrigation performance indicators such as *AE* up to 60% and *DU* up to 87%.

Indeed, surge irrigation significantly improved irrigation performance, which could be mainly attributed to the effect of surge dynamics on infiltration process^17^. Compared to continuous irrigation, water advances more rapidly in surge irrigation, and as a result, the difference in infiltration time at the beginning and end of the furrow is minimized that would result in more uniformly infiltrated water. In fact, this is due to effect of surges on *f*_0_ that result in more precise water infiltration^11^. Previous studies have also revealed that irrigation management can influence the *f*_0_ along a furrow that would influence irrigation performance criteria and uniformity^81,82^.

Stream size is the most important factor that can be controlled by farmers, and optimizing this parameter has high impact in improving irrigation performances^2,12,18^. Our results also confirmed this argument that stream size plays an important role in improving irrigation performances. Increasing the stream size and reducing the opportunity time in both continuous and surge irrigation methods decreased *TWR* and increased the *AE* that are in agreement with Xu et al.^14^ and Ojaghlou et al.^28^. Moreover, increasing the furrow length leads to an increase in the number of cycles in surge irrigation that could increase opportunity time, uniformity distribution, and irrigation performance. However, the longer furrow in surge irrigation would also lead to increased cut-off time that makes it a trade-off a challenging issue to implement and manage surge irrigation. Therefore, local experiments are needed to define the optimum stream size, furrow length, and CR to resolve this tarde-off.

### Comparison of SIRMOD and WinSRFR models

SIRMOD and WinSRFR both had excellent accuracies in simulating the field observations such as advance-recession times, runoff volume, and infiltrated water depth under continuous and surge irrigation methods (Tables 5, 6). In the case of surge irrigation, SIRMOD uses infiltration parameters in the first cycle (as a dry and continuous condition), and the third cycle (as wet and intermittent flow condition). However, since WinSRFR benefits from more physically-based approaches for parameterizing the infiltration equations^47,83^, the accuracy of the WinSRFR model was higher than SIRMOD. In this regard, the lower accuracy of SIRMOD in simulating runoff volume under surge irrigation could be attributed to the inability of SIRMOD to simulate the runoff volume under surge irrigation that is also reported by Ismail et al.^35^.

Both SIRMOD and WinSRFR models had high accuracies in simulating irrigation performance indicators for continuous and surge irrigation (Table 7). The development of SIRMOD and WinSRFR models were primarily based on continuous irrigation as the most common furrow irrigation with simpler flow hydraulics compared to surge irrigation^15,28,31^. SIRMOD model has been used extensively in different part of the world due to its longer history in surface irrigation simulation, while the WinSRFR model has been more popular in the USA because of being user-friendly as well as extensive and flexible modeling features^13^. Furthermore, WinSRFR model can analyze, simulate, design, and optimize the irrigation systems, which highlights the higher ability and flexibility of WinSRFR compared to SIRMOD in irrigation assessments. However, comparing the SIRMOD and WinSRFR in simulating the irrigation performance indices (Table 7) revealed that WinSRFR resulted in more consistent simulations under combinations of stream size and furrow length for either of the continuous or surage irrigation. This shows that WinSRFR could be used more reliably and confidently than SIRMOD in planning, desiging, and evaluating different scenarions of furrow irrigation methods. The outstanding feature of the WinSRFR model in choosing and parametrizing diverse types of infiltration models would be a strong reason for the higher performance and consistency of WinSRFR in simulating surge irrigation compared to SIRMOD. This might be, however, due to the improved ability of the WinSRFR model in infiltration parameterization^83,84^. This leads to significantly improved performance in simulation of infiltration depth and other irrigation performance indicators^9,85^. In addition, the WinSRFR model simulates the surge irrigation process progressivly since the water inflow until the cut-off time. In contrary to WinSRFR model, the SIRMOD model is not able to simulate the surge irrigation progressively and continuously beyond the advance time, which reduces the accuracy of the simulation. In general, the results of this study showed that using WinSRFR model could lead to the straightforward and consistent simplification in simulation and evaluation of surge irrigation.

Overall, the results showed that surge irrigation improved irrigation performances and the SIRMOD and WinSRFR models could be applied for precise irrigation design and managements. The results also showed that the WinSRFR model had better performance because it simulates the physical properties of surge irrigation and its infiltration parameters with higher accuracy compared to the SIRMOD model. This reinforces that the irrigation models are strong and reliable analytical tools in evaluating the performance of site-specific irrigation managements. However, the models need to be tested and verified based on local field data before generalizing and upscaling for a larger region. In future researches, it is suggested to increase the accuracy of the WinSRFR model simulations by improving the structure and physical water-soil relationships governing surge irrigation such as infiltration parameters and field geometry.

Since the latest version of the SIRMOD model was introduced in 2005^62^, and no further updates have been released, it is not possible to improve the model performance in surge irrigation simulation. However, the WinSRFR model is regularly updated^58^. Therefore, it is most likely that its future versions will have higher abilitites to simulate surge irrigation as it it becoming a popular water-saving irrigation method^76,86,87^.

## Conclusion

The continuous and surge irrigation strategies were assessed under the combinations of different furrow lengths and stream sizes for calculating the irrigation performance indicators. The field observations proved that surge irrigation increased the application efficiency and distribution uniformity and decreased deep percolation and runoff volume compared to the continuous irrigation, which confirmed our hypothesis for improved irrigation performance under surge irrigation. Reducing the stream size from 0.6 to 0.4 l/s and increasing the furrow length from 70 to 90 m increased irrigation application efficiency and distribution uniformity, which resulted in reduction of deep percolation and tail-water ratio. These observations showed that the highest irrigation performance was obtained with the combination of stream size of 0.4 l/s and furrow length of 90 m.

The SIRMOD and WinSRFR models were used to simulate and assess the field observations of continuous and surge irrigation. The simulations showed that the performance of WinSRFR and SIRMOD were excellent to simulate the advance-recession times, runoff volume, and infiltrated water depth under both irrigation methods for estimating irrigation performance indicators. The results indicated that WinSRFR performed slightly better than SIRMOD for simulating application efficiency (irrigation management performance) and distribution uniformity (irrigation method performance). In general, WinSRFR benefits from a set of advanced infiltration equations, an option for simulating different cycle ratios in surge irrigation (SIRMOD just simulates the CR = 0.5), and continuously regular development and revision by the model developer.

In conclusion, our study showed that shifting from continuous irrigation to surge irrigation can improve irrigation performance. In this regard, it is necessary to determine the appropriate combinations of stream size, furrow lengths, and cutoff time by using the surface irrigation models. Therefore, it is widely possible to model diverse irrigation and field management scenarios to improve irrigation performance indicators to save water and reduced water loss in irrigated agricultural systems.

## Data Availability

All data generated or analysed during this study are included in this published article.
